# Immune checkpoint inhibitors in colorectal cancer: limitation and challenges

**DOI:** 10.3389/fimmu.2024.1403533

**Published:** 2024-06-11

**Authors:** Suying Yan, Wanting Wang, Zhiqiang Feng, Jun Xue, Weizheng Liang, Xueliang Wu, Zhiquan Tan, Xipeng Zhang, Shuai Zhang, Xichuan Li, Chunze Zhang

**Affiliations:** ^1^ School of Integrative Medicine, Tianjin University of Traditional Chinese Medicine, Tianjin, China; ^2^ Department of General Surgery, The First Affiliated Hospital of Hebei North University, Zhangjiakou, China; ^3^ Central Laboratory, The First Affiliated Hospital of Hebei North University, Zhangjiakou, China; ^4^ Institute of Cancer, The First Affiliated Hospital of Hebei North University, Zhangjiakou, China; ^5^ Department of Scientific and Technical Information, Tianjin Union Medical Center, Tianjin, China; ^6^ Department of Colorectal Surgery, Tianjin Union Medical Center, Tianjin, China; ^7^ The Institute of Translational Medicine, Tianjin Union Medical Center of Nankai University, Tianjin, China; ^8^ Tianjin Institute of Coloproctology, Tianjin, China; ^9^ Tianjin Key Laboratory of Animal and Plant Resistance, College of Life Sciences, Tianjin Normal University, Tianjin, China

**Keywords:** immune checkpoint inhibitors, colorectal cancer, microsatellite instability, drug resistance, biomarker

## Abstract

Colorectal cancer exhibits a notable prevalence and propensity for metastasis, but the current therapeutic interventions for metastatic colorectal cancer have yielded suboptimal results. ICIs can decrease tumor development by preventing the tumor’s immune evasion, presenting cancer patients with a new treatment alternative. The increased use of immune checkpoint inhibitors (ICIs) in CRC has brought several issues. In particular, ICIs have demonstrated significant clinical effectiveness in patients with MSI-H CRC, whereas their efficacy is limited in MSS. Acquired resistance can still occur in patients with a positive response to ICIs. This paper describes the efficacy of ICIs currently in the clinical treatment of CRC, discusses the mechanisms by which acquired resistance occurs, primarily related to loss and impaired presentation of tumor antigens, reduced response of IFN-λ and cytokine or metabolic dysregulation, and summarizes the incidence of adverse effects. We posit that the future of ICIs hinges upon the advancement of precise prediction biomarkers and the implementation of combination therapies. This study aims to elucidate the constraints associated with ICIs in CRC and foster targeted problem-solving approaches, thereby enhancing the potential benefits for more patients.

## 1 Introduction

More than 1.9 million new cases of colorectal cancer (CRC) and 904,000 deaths were estimated to occur in 2022, representing close to one in 10 cancer cases and deaths. Overall, colorectal cancer ranks in third place in terms of incidence but second in terms of mortality (1). In 2022, China recorded a total of 517,100 new cases of CRC, placing it second in terms of incidence and fourth in terms of mortality among all types of cancers (2). Around 20% of individuals diagnosed with CRC have metastases at the time of diagnosis, and this percentage has remained constant over the previous 20 years (3). The primary treatment for unresectable metastatic CRC (mCRC) is systemic therapy (cytotoxic chemotherapy, biologic therapy such as antibodies to cellular growth factors, immunotherapy, and their combinations) (4). However, the five-year survival rate for mCRC remains poor, at around 14% (5). The development of immunotherapy, especially immune checkpoint inhibitors (ICIs), provides novel therapeutic options for mCRC.

Currently used immune checkpoint inhibitors (ICIs) can be categorized into programmed cell death protein 1/programmed cell death 1 ligand 1 (PD-1/PD-L1) inhibitors and cytotoxic T-lymphocyte-associated protein 4 (CTLA-4) inhibitors, based on the specific immunosuppressive receptor they target. ICIs have demonstrated effectiveness in clinical trials, with nivolumab, pembrolizumab, and ipilimumab approved by the FDA for CRC patients with microsatellite instability-high or mismatch repair deficient (MSI-H/dMMR) (6–8).

MMR system dysfunctions or mutations (dMMR) cause DNA mutations to accumulate (9), which produce enough tumor neoantigens to enhance tumor immunogenicity and trigger a potent T-cell and tumor immune response that allows MSI-H/dMMR CRC patients to respond to ICI therapy (9, 10). MSI-H/dMMR CRC exhibited elevated numbers of CD8^+^ T cells and Th1 cell infiltration, and there was a notable upregulation of T cell suppressor ligands, including PD-L1, as well as the B80 family of CD86 and CD7 (11, 12). Upon binding to the co-inhibitory receptors, ICIs exploit the existing inflammatory microenvironment by inhibiting T-cell inhibitor signaling, thereby rendering cancer cells susceptible to cytotoxic injury. In contrast, CRC with microsatellite instability-low (MSI-L) exhibited a lack of immunostimulatory neoantigen production and a relative decrease in the expression of immunosuppressive ligands (13). MSI-H/dMMR is present in only 5% of patients with mCRC (14, 15). This means that the clinical application of ICIs in CRC is considerably restricted. Even patients with MSI-H/dMMR may ultimately develop ICI resistance and suffer illness progression. In addition, adverse effects from ICI therapy, most notably over-immunization of systemic organs, have limited its clinical application (16). The development of predictive biomarkers and the refinement of combination therapy strategies may be both an opportunity and a challenge for ICI therapy to expand the beneficiary population and improve efficacy.

## 2 Clinical trials of ICIs in CRC

ICIs show significant efficacy in MSI-H/dMMR CRC patients (17). Currently, anti-PD-1 monoclonal antibody is the used most in clinical practice, followed by anti-CTLA-4 monoclonal antibody. Specific drug combinations and clinical trials are listed below according to target classification:

### 2.1 PD-1

The anti-PD-1 monoclonal antibodies currently approved by the FDA for clinical application are nivolumab and pembrolizumab. In the KEYNOTE-164 trial, 124 patients with MSI-H/dMMR CRC (61 in cohort A and 63 in cohort B) had a median follow-up of 31.3 and 24.2 months (range, 0.1–27.1 months), with objective remission rates (ORR) of 33% (95% CI, 21% to 46%) and 33% (95% CI, 22% to 46%) (18). In evaluating the efficacy of pembrolizumab in patients with advanced dMMR cancers of different tumor types, 11 patients with MSI tumors and 21 patients with MSS-refractory tumors had an ORR of 0% (95% CI, 0–20) and a PFS of 11% at 20 weeks. (19). Another study included patients with advanced refractory PD-L1-positive colon or rectal cancer regardless of MSI status, with a final median follow-up of 5.3 months, and the majority of patients (n = 15, 65%) experienced disease progression. One patient with MSI-high CRC (4%) experienced partial remission (20).

### 2.2 PD-1 + CTLA-4

Nivolumab is used alone or with ipilimumab to treat MSI-H or dMMR cancer that has spread to other parts of the body and got worse after treatment with a fluoropyrimidine, oxaliplatin, and irinotecan hydrochloride (21). According to the Checkmate-142 study, 23 of 74 patients treated with nivolumab (3 mg/kg every two weeks) achieved an objective response, and 68.9% had disease control for ≥ 12 weeks (7). Pembrolizumab is used to treat MSI-H or dMMR cancer that has spread to other parts of the body or cannot be removed by surgery (21). The immune-related ORR and immune-related progression-free survival rates were 40% (4 of 10 patients) and 78% (7 of 9 patients), respectively, for dMMR colorectal cancers and 0% (0 of 18 patients) and 11% (2 of 18 patients) for pMMR colorectal cancers (22).

In addition, the trial on botensilimab in combination with balstilimab found a median follow-up of 6.4 months (range 1.6–29.5), an ORR of 22% (95% CI, 12–35), and a disease control rate (DCR) of 73% (95% CI, 60–84), which did not meet the median duration of remission. 12-month overall survival (OS) was 61% (95% CI, 42–75), with a median OS not met (23).

### 2.3 PD-1 + LAG3

Lymphocyte Activation Gene-3 (LAG-3), also known as CD223, has the primary function of negatively regulating T cell function and is a member of the immunoglobulin superfamily. LAG-3 molecule negatively regulates T cells and plays a vital role in maintaining the homeostasis of the immune system and promoting tumor immune escape. As a new target, LAG-3 has excellent potential in tumor immunotherapy. Clinical studies on LAG-3 inhibitors are still at a relatively early stage. In the NCT02720068 trial, the median follow-up was 5.8 months in the favicelizumab arm and 6.2 months in the favicelizumab combined with the pembrolizumab arm (24). Another trial combining BI 754111 and BI 754091 showed that of 40 patients with advanced solid tumors with MSS mCRC, 3 (7.5%) patients achieved partial remission (PR), and 11 (27.5%) patients had stable disease (SD) in optimal remission (25). Overall, promising clinical outcomes have been observed thus far with the combination of LAG-3 and PD-1 inhibitors.

### 2.4 PD-L1

The PD-L1 blocker atezolizumab demonstrated better efficacy in the NCT02788279 trial, with a median follow-up of 7.10 months (6.05–10.05) (26). Another trial of the blocker durvalumab included 30 cases for MSI-H/dMMR and 3 cases of POLE mutant MSS CRC, with a median follow-up of 11.2 months (95% CI: 7.3–15.0) and an ORR of 42.4% (95% CI: 25.5–60.8) (27).

### 2.5 PD-L1 + CTLA-4

A single-arm Phase 1b/2 MEDITREME trial evaluated the safety and efficacy of durvalumab in combination with tremelimumab in combination with mFOLFOX6 chemotherapy as first-line treatment in 57 patients with unresectable metastatic CRC with RAS mutations. The Phase 2 primary efficacy goal in patients with MSS tumors was met with a 3-month PFS of 90.7% (95% confidence interval (CI): 79.2–96%). For the secondary objective, the response rate was 64.5%; the median PFS was 8.2 months (95% CI: 5.9–8.6); and overall survival was not achieved in patients with MSS tumors (28).

Despite the clinical efficacy of ICIs, it is important to note that the potential benefits of this therapy are limited to a specific population, that is, patients with MSI-H/dMMR. In addition, the identification of issues such as drug resistance and adverse events has hampered the therapeutic application of ICIs.

## 3 The limitation of ICI therapy in CRC

The response rates to ICI treatment exhibit significant variability among different subgroups of CRC patients. Based on the hypothesis, dMMR patients will benefit from ICIs, whereas pMMR patients will not (22). Nonetheless, dMMR/MSI-H is uncommon (approximately 20% of CRC and 5% of mCRC patients) (14, 15). Even patients who initially respond to ICIs may ultimately develop acquired drug resistance and suffer disease progression. In conclusion, only a minority of patients acquire a long-term and durable response to ICIs, while most patients develop resistance (29). Immune-related adverse events that follow ICI therapy have also limited its clinical application.

### 3.1 Limited population responsive to ICIs

ICIs have exhibited significant clinical efficacy in CRC patients with MSI-H, while limited efficacy in CRC patients with MSS/MSI-L. In a phase II clinical trial that aimed to evaluate the effectiveness of pembrolizumab in CRC patients with MSI-H and MSS, the rates of immune-related OR and immune-related PFS at 20 weeks, were found to be 40% and 78%, respectively, in the MSI-H cohort. In contrast, the corresponding percentages in the MSI-L cohort were 0% and 11%. Furthermore, the MSI-H cohort had a greater concentration of CD8^+^ cells (22). In clinical trials in the MSI-L CRC population, it revealed that following 8 cycles of treatment with pembrolizumab and maraviroc, the median PFS was 2.10 months, the median OS was 9.83 months (30). Additionally, when pembrolizumab was administered with ibrutinib, the median PFS was 1.4 months, and the median OS was 6.6 months (31).

Trials targeting the MSI-H CRC population have yielded improved clinical outcomes. In the CheckMate-142 trial, the treatment combination of Nivolumab plus ipilimumab had 9- and 12-month PFS rates of 76% and 71%, respectively, and 9- and 12-month OS rates of 87% and 85%, respectively (8). The subsequent tremelimumab treatment combination of Nivolumab plus low-dose ipilimumab had 24-month PFS and OS rates of 74% and 79%, respectively (32). In the KEYNOTE-164 trial, Pembrolizumab treatment in patients with two prior lines of standard therapy was associated with an OS of 31.4 months (18). The clinical trials that have produced outcomes for ICIs in MSI-H and MSS CRC are presented in **Table 1**.

**Table 1:** The current clinical trails of ICIs in MSI-H and MSS CRC.

NCT Number	Interventions	Enrolment	Primary endpoint	Outcome
MSS+MSI-H				
NCT01876511 (22)	pembrolizumab	32 CRC patients with pMMR or dMMR	ORR PFS	dMMR: ORR = 40%; PFS = 78% pMMR: ORR = 0%; PFS = 11%
MSI-H				
NCT02460198 (18)	pembrolizumab	124 metastatic MSI-H/dMMR CRC patients treated with ≥ 2 prior lines of standard therapy or ≥ 1 prior line of therapy	ORR	≥ 2 prior lines: ORR = 33% ≥ 1 prior lines: ORR = 33%
NCT02563002 (33)	pembrolizumab or chemotherapy	307 patients with metastatic MSI-H/dMMR CRC who had not previously received treatment	PFS OS	Pembrolizumab: PFS = 16.5 months chemotherapy: PFS = 8.2 months (follow-up time: 32.4 months)
NCT02060188 (7, 8, 32)	nivolumab plus low-dose ipilimumab nivolumab plus ipilimumab nivolumab	Eastern Cooperative Oncology Group (ECOG) performance status of 0 to 1 Histologically confirmed recurrent or metastatic colorectal cancer Microsatellite instability expression detected by an accredited laboratory Participants enrolled into the C3 Cohort must have not had treatment for their metastatic disease	ORR	nivolumab plus low-dose ipilimumab:ORR=69%(95% CI, 53 to 82) DCR=84%(95% CI, 70.5 to 93.5) nivolumab plus ipilimumab: ORR=55% (95% CI, 45.2 to 63.8) DCR≥ 12 weeks = 80% nivolumab: ORR=31.1% (95% CI, 20.8 to 42.9) DCR≥ 12 weeks = 68.9%
NCT03350126 (34)	ipilimumab nivolumab	57 patients with 1.In ICH, the extinction of MLH1 (+/- PMS2), or MSH2 (+/- MSH6), or MSH6, or PMS2 alone for inclusion (dMMR), 2.In PCR, BAT25, BAT26, NR21, NR24, and NR27. Only tumor samples with ≥2 instable markers for inclusion (MSI-H).	DCR	DCR=86%
NCT02715284 (35)	dostarlimab	69 patients with Status of tumor MMR/MSI: needs to be determined by MMR IHC results.	ORR	ORR=36.2%
NCT03150706 (36)	avelumab	33 patients with Mismatch repair deficient or microsatellite instable (defined below), or POLE mutated tumors	ORR	ORR=24.2%
NCT03186326 (37)	avelumab	132 patients with MSI-H determined Mutational status RAS and BRAF	PFS	PFS=12 months
NCT02227667	durvalumab	36 patients with Microsatelite-high colorectal cancer; Locally advanced or metastatic CRC	ORR	ORR=22%
MSS				
NCT03631407	vicriviroc (150 mg or 250 mg) in combination with pembrolizumab (200 mg)	41 participants with advanced/metastatic MSS CRC	ORR	Vicriviroc (150 mg) + Pembrolizumab (200 mg):ORR = 5% Vicriviroc (250 mg) + Pembrolizumab (200 mg):ORR = 5%
NCT03274804 (30)	pembrolizumab and maraviroc	20 patients received pembrolizumab and maraviroc, followed by pembrolizumab monotherapy.	feasibility rate	feasibility rate = 94.7%
NCT02981524 (38)	GVAX/Cy in combination with pembrolizumab	17 patients with pMMR	ORR	no objective responses PFS = 82 days median OS = 213 days
NCT02860546 (39)	trifluridine/tipiracil plus nivolumab	18 patients with MSS mCRC	ORR	No patient achieved a tumor response Median PFS = 2.2 months
NCT04126733	regorafenib plus nivolumab	94 patients with MSS/pMMR CRC	ORR	ORR = 7%
NCT03271047	nivolumab+binimetinib (+ Ipilimumab)	21 patients with MSS mCRC	Incidence of DLTs	Nivolumab + Binimetinib: incidence of DLTs = 11% Nivolumab + Ipilimumab + Binimetinib: incidence of DLTs = 18.2%
NCT04166383	vascular biogenics (VB)-111 and nivolumab	14 patients with MSS CRC that has spread to the liver	safety and tolerability BOR	Complete Response: 0% Partial Response: 0% Progressive Disease: 84.6% Stable Disease: 15.4%
NCT03007407	durvalumab plus tremelimumab	21 Patients with MSS mCRC progressing on chemotherapy following palliative hypofractionated radiation	ORR	ORR = 9.52%
NCT03206073 (40)	PexaVec + durvalumab (+ tremelimumab)	34 patients advanced pMMR mCRC	safety and feasibility	not result in any unexpected toxicities PexaVec + durvalumab: PFS = 2.1 months PexaVec + durvalumab + tremelimumab: PFS = 2.3 months

DLTs, dose-limiting toxicities; BOR, Best Overall Response.

When comparing MSS/MSI-L CRC to MSI-H CRC, it was observed that MSI-H CRC had a greater degree of immune cell infiltration, higher levels of immune-related gene expression, and increased immunogenicity. The potential factors contributing to the variations in the effectiveness of ICIs across CRC with distinct microsatellite stability types are likely associated with the tumor immune characteristics and the immune microenvironment. In contrast to MSS/MSI-L CRC, MSI-H CRC exhibited more infiltration of immune cells, elevated expression of immune-related genes, and increased immunogenicity (41). Specifically, MSI-H CRC is characterized by high levels of CD8^+^ T cell, Th1 cell infiltration and IFN-γ secretion (11, 12). To avoid the process of immune-mediated death inside the inflammatory microenvironment of T-cells, cancer cells exhibit a significant increase in the expression of T-cell inhibitory ligands, such as PD-L1 as well as CD86 and CD7 of the B80 family, which bind the co-suppressor receptors PD-1 and CTLA-4. ICIs exploit the existing inflammatory microenvironment by inhibiting the signaling of T-cell inhibitors, hence rendering cancer cells susceptible to cytotoxic damage. Conversely, MSI-L lacks immunostimulation for neoantigen production and has relatively reduced expression of immunosuppressive ligands (42). In addition, MSI-H CRC also exhibited increased expression of genes associated with antigen presentation, cytolytic activity, and IFN response. The expression levels of chemokines, cytokines, genes linked to the tumor necrosis factor receptor superfamily, and immunological checkpoint genes considerably increased (41). The immunogenicity of cancer cells is a crucial determinant of ICI response. Higher neoantigen burdens are positively correlated with lymphocytic infiltration, tumor-infiltrating lymphocytes (TILs), memory T-cells, and CRC-specific survival, as determined by CRC whole exome sequencing. The median neoantigen load of MSI-H CRC is approximately 20-fold higher than that of MSS/MSI-L, and making them sensitive to immune checkpoint blockade (19, 43).

### 3.2 Acquired drug resistance in ICI therapy

**Figure 1:**
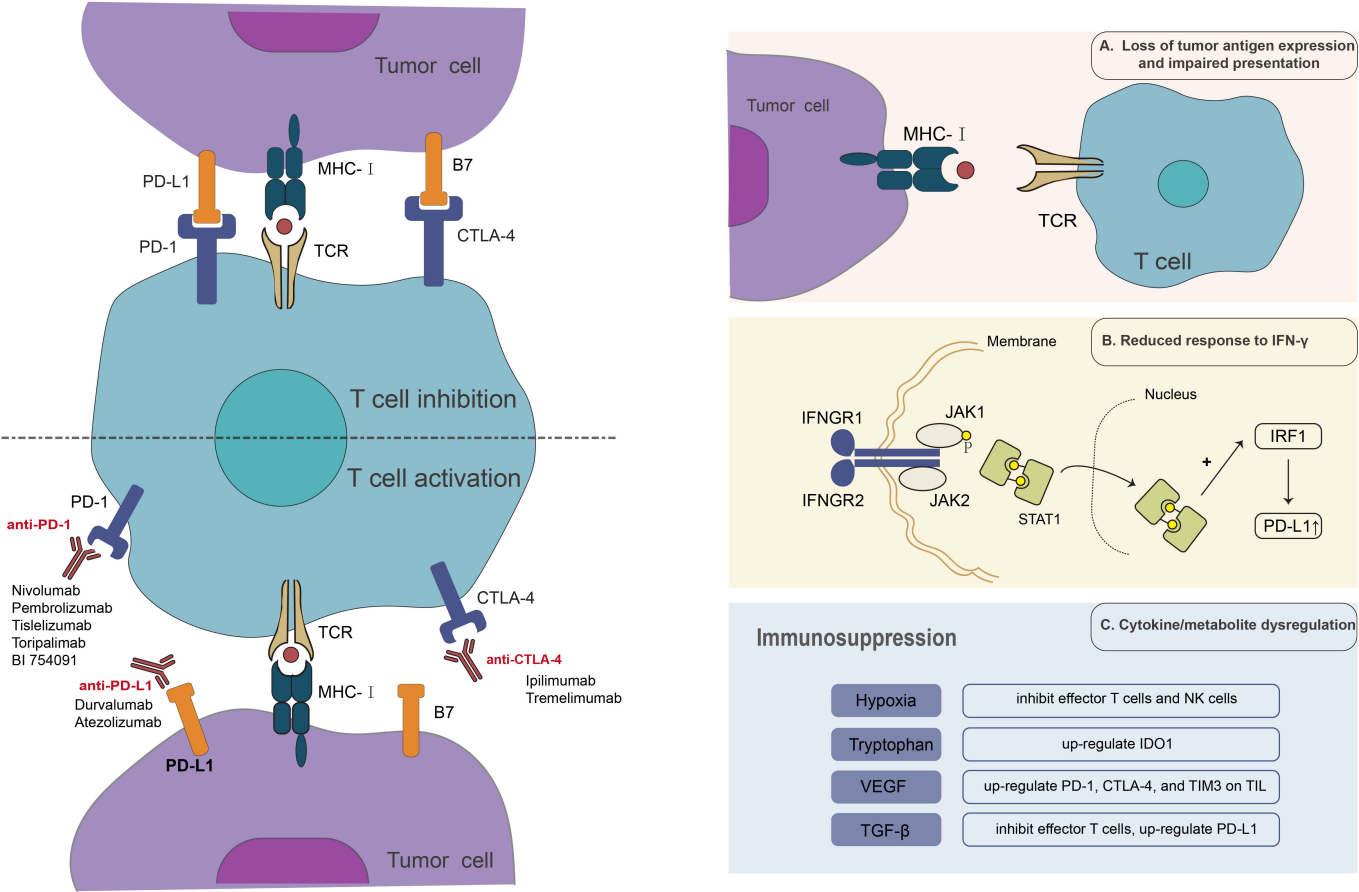
Anti-tumor mechanism of ICIs and mechanism of resistance to ICI therapy.

Acquired drug resistance is considered to be mainly related to the molecular type of the tumor microenvironment (TME) (29). MSI-H/dMMR mCRC tumors display type 1 TME, with a high tumor mutation burden (TMB) and an inflammatory gene profile (29). The type of TME regulates the relationship between tumor cells and the immune system, with elevated TMB levels and an inflammatory genetic profile indicating a prolonged but suppressed immune response (44). MSI-H/dMMR tumors, although most likely to respond to the revitalizing effects of ICI, can still exploit immunosuppressive strategies in the TME signaling pathway to achieve drug resistance (29). Specific immunosuppressive strategies include loss of tumor antigen expression and impaired presentation, reduced response to IFN-γ, and cytokine or metabolite dysregulation (**Figure 1**).

#### 3.2.1 Loss of tumor antigen expression and impaired presentation

Tumor antigens are immune system targets used to recognize cancer cells. This is the initial stage of the anti-tumor immune response and is essential for the anti-tumor effects of ICIs. Loss of tumor antigens renders the immune system incapable of recognizing tumor cells and initiating an immune response in the body. On the other hand, under the strain of anti-tumor immunity, cancer antigens are reduced or lost, a process known as antigen regulation, which permits tumor cells to elude immune recognition and killing. Tumors characterized by elevated mutational loads and neoantigen loads, such as melanoma and non-small cell lung cancer (NSCLC), tend to exhibit more sensitivity to ICIs. Conversely, most CRC patients often demonstrate lower mutational loads (45). In MSI-H CRC, expression of the structural components of the major histocompatibility complex (MHC) is hindered by mutations that disrupt antigen presentation (46). MHC-I displays tumor antigens on the cell surface. The absence or weak expression of MHC-I of tumor cells reduces the presentation of tumor antigens and cannot provide the first signal for T cell activation, resulting in T-cell-resistance in tumor cells (47, 48). β_2_-microglobulin(β_2_M) contributes to the transport and steady expression of MHC on the cell surface. Frameshift deletion of β_2_M disrupts the transport of MHC-I to the cell surface, thereby rendering tumor cells invisible to cytotoxic CD8^+^ T cells and ultimately inducing acquired ICI resistance (49, 50). Studies in CRC have also found that increased β2M mutations are significantly associated with increased infiltration of PD-1-positive T cells and are significantly associated with the MSI phenotype (51, 52). Also, the interference of the original protease processing process, and the function of the transporter that regulates the treatment of the antigen, can destroy the antigen representation of the process (46). To address ICI resistance due to the lack of tumor antigens, tumor vaccines have been used in combination with ICIs. The primary aim of cancer vaccines is to elicit an adaptive immune response against specific tumor antigens, resulting in the regression of tumors (53). In a mouse model, Duraiswamy et al. demonstrated that simultaneous blockade of PD-1 and CTLA-4 in the presence of a GVAX vaccine induced 100% rejection of CT26 colorectal tumors in mice. However, this effect has not yet been validated in clinical trials (54, 55). However, the utilization of this combination therapy is a strategic approach aimed at mitigating the issue of ICI resistance.

#### 3.2.2 Reduced response to IFN-γ

Interferon-γ (IFN-γ) is primarily released by CD8^+^ cytotoxic T cells and CD4^+^ Th1 cells. IFN-γ binds to the IFN-γ receptor (IFNGR), which activates Janus kinases 1 (JAK1) and 2 (JAK2), followed by the recruitment and phosphorylation of STAT1 (56). The complex is transferred to the nucleus, activating the interferon adjustment factor 1(IRF1), and its transcription activity eventually leads to the anti-tumor effect of the aconic-mediated anti-tumor, and the increased PD-L1 expression (57–60). Bioinformatics analysis studies have shown that transcriptional profiles enriched in IFN-γ-responsive genes are positively associated with prognosis and response to anti-tumor immunotherapy (61). In contrast, tumors having a transcriptional profile of inherent anti-PD-1 resistance did not react to anti-PD-1 ICI (62). In a study designed to prospectively predict the response of NSCLC patients to checkpoint inhibitor therapy, patients with elevated IFN-γ levels benefited significantly from ICI therapy (63). JAK1/JAK2 mutations lead to blocked signaling of IFN-γ and consequent lack of PD-L1 expression (59, 64). Analysis of the TCGA database revealed the presence of JAK1 mutations in 10% of CRCs and JAK2 mutations in 12% of CRCs. Whole-exome sequencing of tissue from CRC patients resistant to PD-1 blockade therapy has revealed JAK1 mutations (65). In conclusion, a reduced response to IFN-γ is one of the essential mechanisms of ICI resistance.

#### 3.2.3 Cytokine or metabolite dysregulation

Hypoxia in the tissue microenvironment (TME) enhances the accumulation of extracellular ATP metabolized to adenosine and generates potent immunosuppression (66). On one side, adenosine may inhibit effector T cells and NK cells (66–68). ADP-mediated immunosuppression via adenosine synthesis, on the other side, induces tumor resistance to PD-1/PD-L1 inhibitors (69). Tryptophan catabolism in TME mediates immunosuppression by overexpressing indoleamine 2,3-dioxygenase 1 (IDO1), which can induce IFN-γ (70). The expression of IDO1 is increased following treatment with ICIs, and it is inducible for other checkpoints (71). IDO1 inhibitors shows synergistic effects when combined with ICIs, according to preclinical research, however this has not been confirmed in clinical trials (72). Recent clinical trials have demonstrated, nevertheless, that tryptophan 2,3-dioxygenase (DTO), a key rate-limiting enzyme along with IDTO, has a stronger correlation with the tryptophan-kynurenine pathway, leading to tumor progression and ICI resistance in renal cell carcinoma patients (73).

In addition to the dysregulation of metabolites, tumor cells acquire drug resistance by the overexpression of immunosuppressive cytokines, such as VEGF and TGF-β. VEGF within the tumor microenvironment exerts a down-regulatory effect on adhesion molecules, including ICAM-1 or VCAM-1, and inhibits T-cell trafficking and dendritic cell development. Therefore, it is reasonable to suggest that the administration of antiangiogenic medications may have the capacity to mitigate these occurrences, leading to a synergistic antitumor impact when combined with ICI treatment. This assertion supports in numerous *in vitro* investigations (74). TGF-β is an inhibitory cytokine released by Treg that inhibits effector T cell responses and has been demonstrated to upregulate PD-L1 expression (75, 76).

## 4 Predictive biomarkers for ICIs

Despite the remarkable levels of enduring remission witnessed in cancer immunotherapy, a majority of patients do not experience any therapeutic benefits (primary resistance). In contrast, a subset of individuals who initially react to treatment may later experience a relapse (acquired resistance) (77). The patient heterogeneity in response to immune checkpoint suppression is comparable to the issue of identifying responders and non-responders to traditional first-line neoadjuvant chemotherapy (78). Researchers are looking for biomarkers and personalized genes through which they hope to identify the ideal patient candidates for immunotherapy. We are listing biomarkers that are currently considered to have some predictive value. MSI-H and POLE mutations are regarded as biomarkers, and the POLE mutation is considered a promising marker for enhancing the efficacy of immunotherapy in MSS mCRC patients (79). In addition to this, other markers are also valuable and worth developing. TMB also has high application value as an independent biomarker for ICI treatment. However, determining the critical value of TMB and optimizing its detection method is challenging (80, 81). The use of PD-L1 as a biomarker to guide ICI therapy has not been validated, but it is also true that PD-L1 is one of the most established biomarkers available (82). Achieving reproducibility of PD-L1 assays and developing combination applications of biomarkers may facilitate the use of PD-L1 as a stable biomarker for ICI therapy in CRC patients (83, 84).

### 4.1 Expression of PD-L1

In 2019, the American Society of Clinical Oncology Gastrointestinal Conference viewed the expression of PD-L1 combined positive score ≥ 10 as a potential biomarker for the treatment of advanced esophageal cancer with pembrolizumab (85). The predictive role of PD-L1 have been confirmed in the solid tumors immunotherapy and high PD-L1 expression is associated with clinical benefit. Still, there is no consensus on the performance of PD-L1 as a predictive target. The CheckMate-142 study assessed the effects of nivolumab and PD-L1 on tumor cells or immune cells. As Lu et al. hypothesized, the data demonstrated no link between the expression and the immunotherapy response (8, 86). These disparate outcomes may be attributable to the varied antibodies used, the unequal distribution of PD-L1 in the tumor and mesenchyme, and changes in PD-L1 expression before and after therapy (87–89). PD-L1 is not a stand-alone and comprehensive biomarker. Its main drawbacks are the lack of a universal threshold for PD-L1 expression, insufficient standardization of PD-L1 assays and antibodies, and the spatial and dynamic heterogeneity of PD-L1 expression (90). To maximize the therapeutic potential of PD-1/PD-L1 blockers, predictive biomarkers of therapeutic response need to be identified, new therapeutic strategies need to be developed, and therapeutic strategies for combinations with other agents must be improved (91). Improved prediction of PD-L1 assessment can be achieved by the following methods: assessment of PD-L1 status, assessment of PD-L1 expression kinetics, evaluation of PD-1/PD-L1 proximity, and automated digital pathology algorithms (91). Single biomarkers often lack the sensitivity and specificity to predict response to ICIs reliably. Recent reports have shown that combinations of multiple biomarkers based on TMB, PD-L1 expression, NLR (neutrophil to lymphocyte ratio), or gene expression profiles have greater sensitivity and specificity than single biomarkers in predicting clinical response (92).

### 4.2 Tumor mutational burden

TMB indicates the number of acquired somatic mutations in the coding regions of the cancer cell genome. High somatic mutation loads are a unifying feature of many cancer types for which ICI therapies have proven effective (93). For MSI-H and MSI-L, ORRs were 54.5% and 31.0%, mPFS was 8.3 and 5.6 months, and median OS was NE and 19.8 months, respectively, for patients with TMB≥16 mut/MB, according to the results of the clinical research. In the MSI-L population, PFS was significantly better in patients with TMB≥16 mut/MB than with 10≤TMB<16 mut/MB (p<0.0001) (94). Initially TMB was determined by whole exome sequencing (WES) of tumor DNA and matched normal DNA. More recently validation of targeted NGS assay combinations has begun based on WES data (95). The panels tested to date (F1CDx and MSK-IMPACT) have demonstrated their ability to predict ICI responses (96).

TMB as a biomarker faces two main challenges: first, the predictive limitations of TMB itself. It was found that only hypermutated tumors benefit from ICIs, and MSS tumors with high TMB do not (97). Current FDA approvals granted based on tumor mutational load may be too broad, and immune checkpoint inhibitors should be considered in the context of the cause of high tumor mutational load, not just on the basis of absolute thresholds. Second, there are challenges in methods for detecting TMB, include (1) determining the therapies to which TMB status best informs its response; (2) robust definition of predictive TMB cutpoints; (3) standardization of sequencing panel sizes and designs; and (4) the need for robust technical and informatics rigor to generate precise and accurate TMB measurements across different laboratories (98).

### 4.3 Microsatellite instability

MMR encodes the appropriate mismatch repair protein, and DNA mismatch repair system deficiencies can lead to MSIs, which are categorized into three groups based on the functional integrity of MMR: MSI-H, MSI-L, and MSS (99). MIS-H/dMMR phenotype typically leads high frameshift mutations and the generation of neoantigens, and it stimulates immune recognition and immune cell infiltration which may cause a more robust immune response (100). Therefore, MSI is an effective predictive marker. In the KEYNOTE-062 clinical study, 22 (44%) of 50 patients with TMB ≥10 mut/Mb had MSI-H tumors. However, only 3 (1%) of patients with TMB <10 mut/Mb had MSI-H tumors, indicating that high TMB is frequently associated with MSI-H (101).

### 4.4 POLE mutation

DNA Polymerase Epsilon (POLE) is a crucial enzyme involved in DNA synthesis and repair, and mutations in POLE prevent DNA repair deficiencies and genetic material mistakes from being corrected, resulting in a significant number of mutations (102, 103).

In CRC patients with POLE mutations, which are usually with MSS, elevated numbers of TIL, promoted PD-L1 expression, and upregulated production of cytotoxic T cell markers and effector cytokines indicate heightened tumor immunogenicity. Notably, MSS CRC patients with POLE mutations have a long-term and durable clinical response to ICI treatment (104). This shows that POLE mutations are a promising marker for enhancing the efficacy of immunotherapy in MSS mCRC patients (79).

### 4.5 Tumor infiltrating lymphocyte

TIL is an essential component of TME, and distinct TIL environments correlate with distinct immunotherapy responses, revealing the complexity of the underlying tumor-immune interactions. The number of TILs is a predictor of ICIs’ effectiveness, and a more significant CD28^+^ TIL cell fraction usually means a more favorable treatment outcome (105). Multiple studies have found relationships between TILs, various histological characteristics of the tumor, disease-free survival (DFS), cancer-specific survival (CS), and overall survival (OS) (106, 107). High TILs were a positive predictive factor for colorectal cancer specificity and OS, according to a multivariate study of 76 patients (108).

### 4.6 IFN-γ

IFN-γ induced infiltration of CD8^+^ T cells and NK cells into the TME. Antigen-presenting cells (APCs) and cancer cells are stimulated to produce MHC-I by IFN-γ, which improves antigen identification by CD8^+^ T lymphocytes and leads to the death of cancer cells (109). Nevertheless, loss-of-function mutations and genomic modifications in the IFN-γ signaling pathway and antigen presentation signaling pathway result in cancer immune evasion, and IFN-γ may promote tumor antigen loss and induce tumor immune editing, resulting in tumor progression and recurrence (49, 65, 110). In conclusion, the dynamic and kinetic effects of IFN-γ on immunogenicity and immune evasion may ultimately determine the fate of tumor growth. Accordingly, exposure to persistent IFN-γ signaling can cause tumors to acquire immune resistance and increase the expression of immunosuppressive molecules, and INF-γ merits additional investigation as a potential predictive biomarker for the efficacy of immunotherapy in CRC (111, 112).

### 4.7 The intestinal flora microenvironment

Increasing evidence suggests that the gut microbiome (GM) of immunotherapy-treated colorectal cancer patients is related to anti-cancer immune responses. The interaction between the gut microbiota and the gastrointestinal mucosa influences the local immune response and the systemic innate and adaptive immune responses. Antibiotics administered 60 days before or after the initiation of an ICI are linked to inferior results in several cancer types (113). Multiple bacteria, including *Akkermansia, Faecalibacterium, Clostridium*, and *Bifidobacterium* have been associated to the antitumor effects of PD-L1 inhibitors (114). Additionally, host immune cells can interact directly with particular bacteria, such as *Akkermansia muciniphila*, which increases the efficiency of immunotherapeutic drugs in an IL-12-dependent way by engaging directly with DCs in lymph nodes (115). Bacteroides also directly improve the anti-tumor immune response of Th1 and CD8^+^T cells (116).

## 5 Combination of ICIs with other therapies

Up to 95% of patients with MSS/pMMR CRC are unlikely to benefit from a single immunotherapy treatment. Compared to MSI-H patients, patients with MSS mCRC had considerably reduced numbers of cytotoxic cells, CD8^+^, Th1, Th2, and T cell markers. Furthermore, there was a striking difference between the proportion of MSI and MSI-H patients in the prevalence of TMB, missense or frameshift mutations, and the number of novel tumor epitopes. Numerous clinical trials have assessed the efficacy and practicability of immunotherapy in combination with other treatments (7, 117, 118).

ICI therapy combined with chemotherapy is a viable treatment strategy. The NCT03388190 study investigated repeated sequential oxaliplatin chemotherapy (FLOX) in combination with nivolumab and FLOX monotherapy for MSS mCRC. Results showed mPFS of 6.6 months (range 0.5–20) and ORR of 46.3% at 8 months in the FLOX + Nivolumab group. This suggests that FLOX therapy can transform MSS into an immunogenic state, allowing lasting disease control in patients with untreated advanced disease where surgery is contraindicated after ICB therapy (119). Similarly, dual immunotherapy was predicted, with the NCT02870920 research demonstrating considerably prolonged OS (6.6 months vs. 4.1 months) and increased disease control rate (DCR) (22.6% vs. 6.6%) in the dual immunization group compared to the control group while no extension of PFS (1.8 months vs. 1.9 months). Moreover, the study indicated that patients with high TMB benefited more from dual immunotherapy (120). The NCT04017650 research also confirmed the efficacy of dual immunotherapy. The combination of encorafenib, cetuximab and nivolumab had an ORR of 45%, a DCR of 95%, a mPFS of 7.3 months and a mOS of 11.4 months (121).

Anti-angiogenic treatment enhances TME, enhances and activates effector immune cells, reduces immunosuppressive cells, and alleviates immunosuppression, which is essential for the synergistic effect of immunotherapy. Refractory MSS CRC was treated with nivolumab and regorafenib in the NCT03406871 trial. ORR (28%), mPFS (7.8 months), one-year PFS rate (41.7%), and one-year OS rate (68.0%) were considerably more significant than in prior trials, suggesting immunotherapy combined with anti-angiogenic medicines may have potential benefits (122).

TGF-β inhibitors can reverse immune resistance to immune sensitization, according to preclinical and clinical investigations, and other ongoing or future clinical trials are exploring the possibility of activating inactive tumors to turn “cold” tumors into “hot” tumors, which holds promise for immunotherapy in large numbers of patients with MSS (123). Cancer vaccines may induce cytotoxic anti-tumor immune responses to a range of tumor-specific antigens, and current clinical trials are evaluating the combination of cancer vaccinations with ICIs in CRC patients (124).

## 6 Conclusion

The clinical application of ICIs is currently garnering broad interest. As a novel treatment method different from radiation and chemotherapy, their availability gives hope to some cancer patients, particularly those suffering from melanoma and non-small cell carcinoma. The application of ICIs in colorectal cancer has shown promising results in several clinical trials conducted individually or in combination. Unfortunately, ICIs benefit only a tiny proportion of MSI-H/dMMR CRC patients, and the efficacy of immunotherapy alone for MSS/pMMR CRC has been disappointing, with no approved drugs to date. Even in MSI-H tumors, resistance due to deletion of tumor antigen expression and impaired presentation, reduced response to IFN-γ, and dysregulation of cytokines or metabolites have hampered the use of ICIs in CRC. Thus, addressing resistance to ICIs is critical to improving immunotherapy outcomes in the CRC patient population, and further research is needed to optimize the use of ICIs in colorectal cancer. Strategies to overcome drug resistance can be approached by developing combination therapies targeting multiple pathways, repurposing existing drugs, and developing new drugs to evade resistance mechanisms. We continue to believe that to maximize the advantages of immunotherapy for CRC, it is critical to advance the development of more predictive biomarkers or refine existing biomarkers. This will facilitate the standardization of ICI treatments and enable more patients to benefit from them. The application of biomarkers to guide the treatment of different diseases is an essential step in putting precision medicine into practice. Ideal biomarkers should have high specificity and sensitivity, a wide range of applications, easy sampling and measurement, and standardized detection methods. However, the biomarkers currently known to us do not meet these requirements. It may be possible to maximize the utility of biomarkers by combining applications and improving detection methods. In addition, with the rapidly expanding indications for ICIs, it has become increasingly important to prevent immunotherapy-related adverse effects, although ICIs are relatively less toxic. In conclusion, despite many challenges, ICIs have changed the landscape of colorectal cancer treatment. Ongoing research and clinical trials are essential to address these current obstacles, and ICIs will have an bright future.

## Author contributions

SY: Writing – review & editing, Writing – original draft. WW: Writing – review & editing, Writing – original draft. ZF: Writing – original draft. JX: Writing – review & editing. WL: Writing – review & editing. XW: Writing – review & editing. ZT: Writing – review & editing. XZ: Writing – review & editing. SZ: Writing – review & editing. XL: Writing – review & editing. CZ: Funding acquisition, Writing – review & editing.
